# Discovery and Characterization of the Biflavones From *Ginkgo biloba* as Highly Specific and Potent Inhibitors Against Human Carboxylesterase 2

**DOI:** 10.3389/fphar.2021.655659

**Published:** 2021-05-18

**Authors:** Yun-Qing Song, Rong-Jing He, Dan Pu, Xiao-Qing Guan, Jin-Hui Shi, Yao-Guang Li, Jie Hou, Shou-Ning Jia, Wei-Wei Qin, Sheng-Quan Fang, Guang-Bo Ge

**Affiliations:** ^1^ Department of Gastroenterology, Yueyang Hospital of Integrated Traditional Chinese and Western Medicine, Shanghai University of Traditional Chinese Medicine, Shanghai, China; ^2^ Institute of Interdisciplinary Integrative Medicine Research, Shanghai University of Traditional Chinese Medicine, Shanghai, China; ^3^ Department of Biotechnology, College of Basic Medical Sciences, Dalian Medical University, Dalian, China; ^4^ Qinghai Hospital of Traditional Chinese Medicine, Xining, China; ^5^ Department of Pharmacy & Worldwide Medical Center, Huashan Hospital, Fudan University, Shanghai, China

**Keywords:** biflavones, inhibition, specificity, irinotecan-induced diarrhea, human carboxylesterase 2 (CES2)

## Abstract

Human carboxylesterase 2 (CES2), one of the most abundant hydrolases distributed in the small intestine, has been validated as a key therapeutic target to ameliorate the intestinal toxicity caused by irinotecan. This study aims to discover efficacious CES2 inhibitors from natural products and to characterize the inhibition potentials and inhibitory mechanisms of the newly identified CES2 inhibitors. Following high-throughput screening and evaluation of the inhibition potency of more than 100 natural products against CES2, it was found that the biflavones isolated from *Ginkgo biloba* displayed extremely potent CES2 inhibition activities and high specificity over CES1 (>1000-fold). Further investigation showed that ginkgetin, bilobetin, sciadopitysin and isoginkgetin potently inhibited CES2-catalyzed hydrolysis of various substrates, including the CES2 substrate-drug irinotecan. Notably, the inhibition potentials of four biflavones against CES2 were more potent than that of loperamide, a marketed anti-diarrhea agent used for alleviating irinotecan-induced intestinal toxicity. Inhibition kinetic analyses demonstrated that ginkgetin, bilobetin, sciadopitysin and isoginkgetin potently inhibited CES2-catalyzed fluorescein diacetate hydrolysis *via* a reversible and mixed inhibition manner, with *K*_*i*_ values of less than 100 nM. Ensemble docking and molecular dynamics revealed that these biflavones could tightly and stably bind on the catalytic cavity of CES2 *via* hydrogen bonding and *π*-π stacking interactions, while the interactions with CES1 were awfully poor. Collectively, this study reports that the biflavones isolated from *Ginkgo biloba* are potent and highly specific CES2 inhibitors, which offers several promising lead compounds for developing novel anti-diarrhea agent to alleviate irinotecan-induced diarrhea.

## Introduction

Mammalian carboxylesterases (CES) are key members of serine hydrolases which play key roles in activation of ester prodrugs and hydrolytic metabolism of a variety of xenobiotics bearing ester bond(s) (Hosokawa, 2008; Hatfield et al., 2016). Human carboxylesterase 1 (CES1) and human carboxylesterase 2 (CES2) as two predominant CES isoforms that have been extensively investigated over the past few decades. The CES1 is primarily expressed in the liver, while CES2 is mainly expressed in the small intestine (Wang D. et al., 2018). The clinically drugs including temocapril, oseltamivir, and sacubitril have been reported as the CES1 substrate drugs, while CES2 has been revealed as a key enzyme responsible for the activation of several important anti-tumor prodrugs, such as irinotecan and capecitabine (Potter et al., 1998; Quinney et al., 2005; Shi et al., 2006; Ohura et al., 2011; Shi et al., 2016; Chen et al., 2018). It has been reported that CES2 participated in approximately 99% of the total conversion of irinotecan into SN-38 in the human small intestine (Humerickhouse et al., 2000; Charasson et al., 2004). Increasing evidence has indicated that co-administration with potent CES2 inhibitors may ameliorate irinotecan-induced severe diarrhea *via* blocking the overproduction of SN38 (the cytotoxic product of CPT-11) in human small intestine (Slatter et al., 1997; Hecht, 1998; Hicks et al., 2009). Currently, loperamide (LPA, a marketed anti-diarrhea agent) has been used for alleviating irinotecan-induced intestinal toxicity in clinical settings (Tobin et al., 2005; Baker, 2007). However, the moderate CES2 inhibition potency, as well as the potential adverse effects of LPA (such as headache, dizziness, constipation, nausea and flatulence), strongly limited the long-term administration of this anti-diarrhea agent (Hanauer, 2008; Eggleston et al., 2017; Miller et al., 2017; White, 2019). Therefore, it is urgent and necessary to find more efficacious CES2 inhibitors to reduce the excessive hydrolysis of CPT-11 into its toxic product SN-38 in the intestinal tract.

**FIGURE 1:**
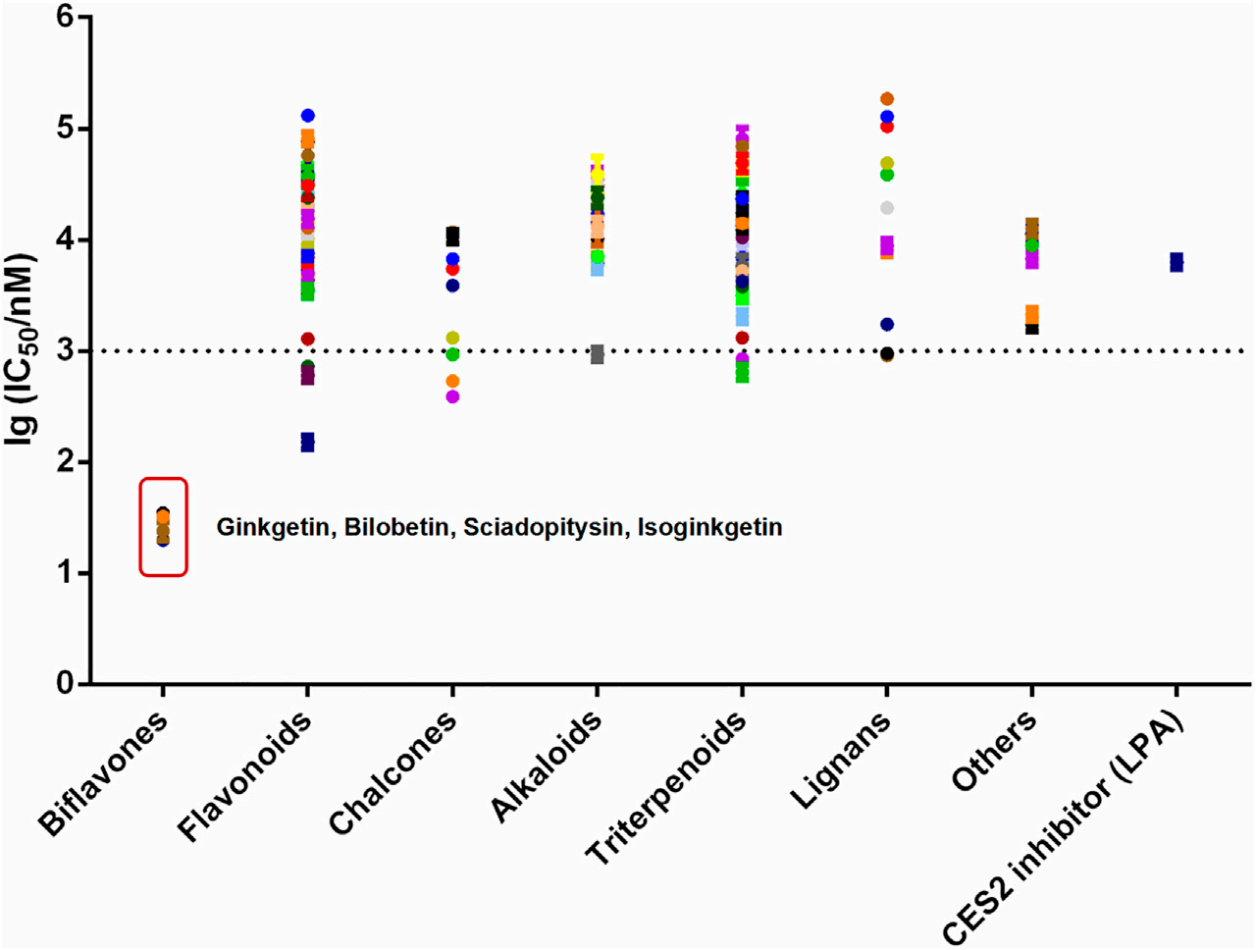
The lg (IC_50_/nM) of more than 100 kinds of natural products and positive inhibitor (LPA) on CES2. All data were shown as mean ± SD of triplicate assays.

**FIGURE 2:**
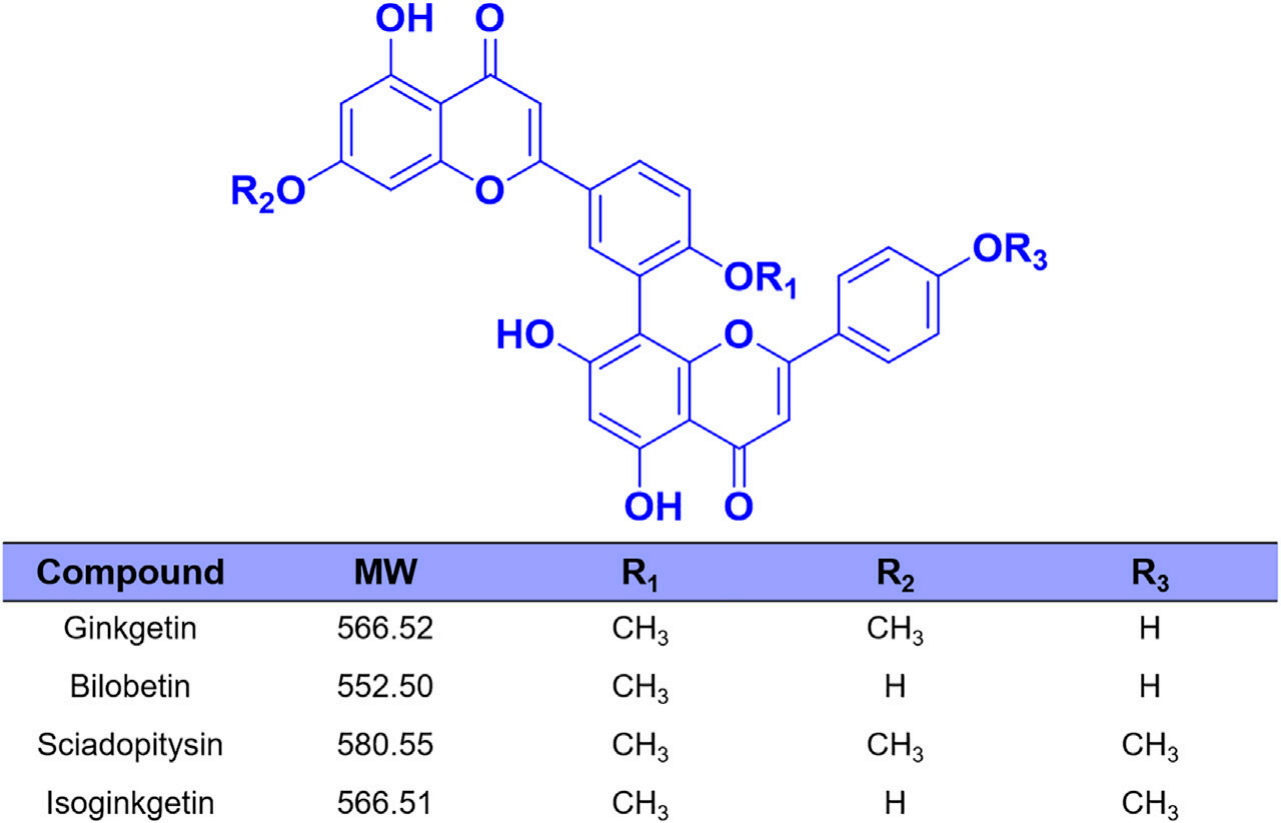
The chemical structures of four biflavones from *Ginkgo biloba*.

Over the past few decades, with the help of the highly specific substrates (including drug substrates and optical substrate) for CES2, a wide variety of natural products (such as tanshinones, triterpenoids and flavonoids) with strong to moderate CES2 inhibition activities were discovered (Zhu et al., 2015; Zou et al., 2016; Weng et al., 2018; Zhao et al., 2020). However, most reported naturally occurring CES2 inhibitors are moderate inhibitors (IC_50_ values are at the micromole levels), which motivates us to find more efficacious CES2 inhibitors. Recently, we have carried out high-throughput screening campaign to evaluate the inhibitory potentials of more than one hundred natural products on CES2. Among all tested natural products, four biflavones (ginkgetin, bilobetin, sciadopitysin and isoginkgetin) isolated from *Ginkgo biloba* leaves were found with the most potent CES2 inhibition activities (Figure 1 and Figure 2). It is well-known that the biflavones isolated from *Ginkgo biloba* leaves have been reported with many beneficial effects, such as anti-oxidative and anti-inflammatory, as well as anti-cancer and potential chemopreventive effects (Chang et al., 2018; Chen et al., 2019; Tao et al., 2019; Souza et al., 2020). Thus, the biflavones hold great promise for alleviating irinotecan-induced intestinal toxicity. Unfortunately, the inhibition potentials and inhibitory mechanisms of the biflavones against CES2 have not been well-characterized yet.

This study aimed to assess the inhibition potentials and specificity of four biflavones isolated from *Ginkgo biloba* against CES2, as well as to characterize the inhibitory mechanisms of these biflavones against CES2. For these purposes, a set of inhibition assays were used to assess the inhibition potentials and specificity of four biflavones against CES2, inhibition kinetic analyses, ensemble docking and molecular dynamics simulations were used to explore the action mechanisms of these four biflavones against CES2. All these studies will be advantageous for deciphering the interactions of four biflavones with CES2 inhibition, while the key findings present here will be promising to develop novel CES2 inhibitors for ameliorating CES2-associated drug toxicity.

## Materials and Experimental

### Chemicals and Reagents

Ginkgetin, bilobetin, sciadopitysin and isoginkgetin were ordered from Shanghai Standard Biotech Co., Ltd. (Shanghai, China). Loperamide (LPA) and fluorescein diacetate (FD) were purchased from TCI (Tokyo, Japan). Irinotecan (CPT-11) and corresponding active hydrolytic metabolite SN-38 were obtained from Alfa Aesar (Beijing, China) and HEOWNS (Tianjing, China), respectively. The purities of all tested biflavones were identified by LC-UV and the data indicated that the purities of tested biflavones were higher than 98% totally. The 4-methylumbelliferyl-acetate (4-MUA) was obtained from Sigma Chemical (Poole, Dorset, United Kingdom). Recombinant CES1 (Batch No.150006A), recombinant CES2 (Batch No.153015A). the pooled human liver microsomes from 50 individual donors (HLMs) and Pooled human intestinal microsomes (HIMs, lot no. X02801) were bought from Bioreclamation IVT (Baltimore, MD, United States) and stored at −80°C. The stock solution of each compound (100 mM) was dissolved with dimethyl sulfoxide (LC grade, Tedia, United States of America), which were stored at 4°C. LC grade acetonitrile (Tedia, United States), phosphate buffered saline (0.1 M, pH 6.8 and pH 7.4) and ultrapure water (produced by a Millipore water system) were used in following experiments.

### Enzyme Inhibition Assays

#### Inhibitor Screening Method Using FD as Probe Substrate

The incubation system contains following components (total volume 200 μL): 2 μL DMSO/inhibitor, 2 μL HLMs (2 μg/ml, final concentration), 194 μL buffer PBS (pH = =7.4). Three concentrations of each inhibitor (1 μM, 10 μM and 100 μM) were established for preliminary screening. Two control groups were set up at the same time, one was the inhibitor-free system (adding equal volume of DMSO) represented the 100% enzyme activity, the other was the positive inhibitor (LPA). The incubation system pre-incubated at 37°C for 3 min minutes and 2 μL fluorescent substrate FD (5 μM, final concentration) was added for fluorescence analysis in fully automated fluorescent microplate reader (SpectraMax M4, Molecular Devices) (Wang et al., 2011).

#### Inhibition of CPT-11 Hydrolysis by Four Bioflavones

CPT-11, a known CES2 substrate-drug, was also used to ascertain whether the identified four biflavones displayed strong inhibition on CES2-catalyzed CPT-11 hydrolysis. Experiments were performed in Eppendorf Tubes (EP, total volume 200 μL): 2 μL DMSO/inhibitor, 2 μL HLMs/HIMs (200 μg/ml, final concentration), and 194 μL PBS buffer (pH = 7.4). Two control groups were set up concurrently, an inhibitor-free (adding equal volume of DMSO) system regarded as 100% enzyme activity, and the other one with the positive inhibitor (LPA). The incubation system pre-incubated at 37°C for 3 min followed by adding 2 μL substrate CPT-11 (5 μM, final concentration), after incubated for another 50 min, reactions were terminated with an equal volume of 10% perchloric acid-ice acetonitrile (1:2). The mixture was centrifuged at 20,000 × g, supernatants were collected for follow-up analysis in LC system (Shao et al., 2012). Chromatographic separation was performed on a shim-pack GIST-HP C18 analytical column (50 mm × 2.1 mm, 3 μm particle size), using ammonium acetate (50 mmol/L, PH = 4), acetonitrile as mobile phase. The detection wavelength of CPT-11 was Ex/Em = 368 nm/432 nm, and the detection wavelength of SN38 was 368 nm/535 nm (Shao et al., 2012).

### Specificity of Four Bioflavones Toward CES2

The incubation system contains following components (total volume 200 μL): 2 μL DMSO/inhibitor, 2 μL recombinant CES1 or recombinant CES2 (5 μg/ml, final concentration), 194 μL buffer PBS (pH = 7.4). Two control groups were set up simultaneously, one was no inhibitor system (adding equal volume of DMSO) regarded as 100% enzyme activity, and the other was the positive inhibitor (nevadensin for CES1 and LPA for CES2) (Wang Y. Q. et al., 2018; Abigerges et al., 1994). The incubation system pre-incubated at 37°C for 3 min and 2 μL substrate 4-MUA (100 μM, final concentration) was added for fluorescence analysis in fully automated fluorescent microplate reader (SpectraMax M4, Molecular Devices) (Gabriele et al., 2018).

### Inhibition Kinetic Analyses

The inhibition constant (*K*_*i*_) and the inhibition modes of four potency inhibitors against CES2-catalyzed FD hydrolysis were explored by utilizing a cluster of kinetic assays for FD hydrolysis at the presence of incremental concentrations of each biflavone (Cheng and Prusoff, 1973). The Michaelis-Menten equations was adopted and transformed to render the Lineweaver-Burk plots, while the slopes of Lineweaver-Burk plots were applied to calculate the inhibition constant (*K*_*i*_) values on the basis of kinetic data.

### Ensemble Docking

Due to the lack of crystal structure of CES2, a modeling structure of CES2 was generated using homologous modeling method by SWISS-MODEL (https://swissmodel.expasy.org/) based on the template structure of CES1 (PDB code: 5A7G) with the resolution of 1.48 Å (Bencharit et al., 2003; Andrew et al., 2018). QMEAN scoring function score of -2.30 validated the reliability of structure model (Benkert et al., 2008). In addition, CES1 and CES2 pertain to serine hydrolase family that catalyze the hydrolysis of numerous endogenous substances and xenobiotics (Zou et al., 2018). The catalytic residue serine is positioned in a deep gorge surrounded by multiple hydrophobic residues (Briand et al., 2019). Account for the flexibility of the catalytic pocket, structurally diverse compounds can be hydrolyzed (Zou et al., 2018). Consequently, comprehending the fluctuation was essential for docking calculation, and ensemble docking approach was applied to predict the binding energies and energetically preferred binding modes (Stoddard et al., 2010). Hence, we performed 1 μs molecular dynamics simulations of CES1 and CES2 to extensively sample the proteins’ conformations, and clustered the representative protein conformations with a tolerance of 2.5 Å root-mean-square deviations (RMSDs) using gromos method. As a result, 8 and 13 representative protein structures were obtained for CES1 and CES2, respectively. We presumed that four biflavones may have similar binding modes due to the similar skeletons, therefore, bilobetin with the most potent inhibitory potency for CES2 was selected to investigate the binding mode and selectivity. Taking the representative protein structures as receptors and bilobetin as ligand, molecular docking was conducted with AutoDock Vina (Version 1.1.2) based on Lamarckian genetic algorithm (Morris et al., 1998; Trott and Olson, 2010). Hydrogen atoms were added followed by assigning the Kollman charges. The center of grid box was set to 60 × 60 × 60 Å^3^ with the spacing of 0.375, enclosing the active site. Favorable binding conformations with the lowest binding energy were indicated for the follow-up calculation.

### Molecular Dynamics Simulation

To further investigate the binding stability of the CES2-bilobetin complex, two binding modes with the best binding affinity (predicted by the ensemble docking as mentioned above) were selected as the initial conformations for molecular dynamics simulations by using Gromacs (Version 2019.3) software (Berendsen et al., 1995; Abraham et al., 2015). The force fields and simulation parameters were exploited in terms of previous research (Song et al., 2019a). Trajectory postprocessing and visualization were performed by Gromacs package and VMD (Version 1.9.3) (Humphrey et al., 1996). Finally, the induced-fit equilibrium state was selected as template to dock with the other three biflavones (Koshland, 1995). The detailed interactions against CES2 were further analyzed and compared.

### Statistical Analysis

All data were shown as mean ± SD of triplicate assays. The GraphPad Prism 8.0 was applied to determine the inhibition constants (including IC_50_ and *K*_*i*_ values).

## Results and Discussion

### Determination of IC_50_ Values of 120 Natural Products Against CES2

**FIGURE 3:**
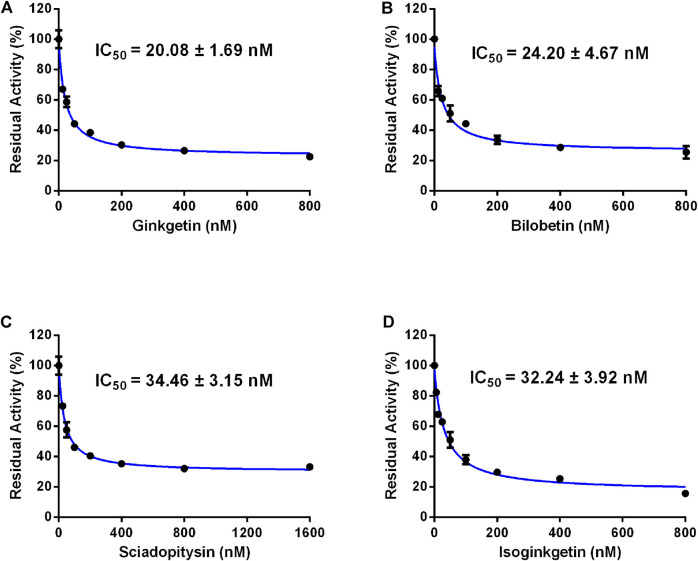
Dose-inhibition curves of ginkgetin **(A)**, bilobetin **(B)**, sciadopitysin **(C)** and isoginkgetin **(D)** on CES2 using FD as the probe substrate in HLMs. All data were shown as mean ± SD of triplicate assays.

In order to discover more efficacious CES2 inhibitors from natural products, more than 100 natural products were collected and their inhibition potentials against CES2 were assayed in HLMs under identical incubation conditions. For each tested compound, at least eight inhibitor concentrations were used to determine the IC_50_ values, by using FD as a fluorescent substrate for CES2. The IC_50_ values of 120 natural products against CES2-catalyzed FD hydrolysis ranged from 20 to 188,000 nM, which were listed in Supplementary Table S1. To compare the inhibition potentials of these natural products in a more intuitive way, the logarithmic transformation of the IC_50_ values in nanomolar were depicted in Figure 1. The dots below the horizontal dashed line represented that these inhibitors displayed relatively strong inhibitory effects on CES2 (less than 1000 nM). Delightfully, it was evident from Figure 1 that four biflavones isolated from *Ginkgo biloba* displayed the most potent inhibition against CES2-catalyzed FD in HLMs, with the calculated IC_50_ values to be 20.08, 24.20, 34.46 and 32.24 nM, for ginkgetin, bilobetin, sciadopitysin and isoginkgetin, respectively (Figures 1, 3). It should be noted that the inhibition potency of these four biflavones were much more potent than that of some reported naturally occurring CES2 inhibitors (such as magnolol, glabridin, Supplementary Table S1) and the positive inhibitor LPA (IC_50_ = 6240 nM, Supplementary Table S1) (Song et al., 2019a; Song et al., 2019b). These finding inspired us to further assess the inhibition potentials of these four biflavones against CES2-catalyzed CPT-11 hydrolysis.

### Inhibition of Four Biflavones Against CES2-Catalyzed CPT-11 Hydrolysis

**FIGURE 4:**
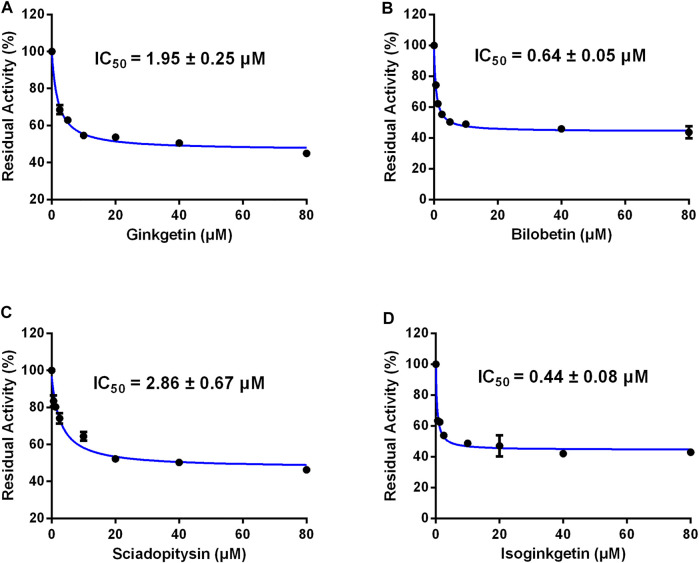
Dose-inhibition curves of ginkgetin **(A)**, bilobetin **(B)**, sciadopitysin **(C)** and isoginkgetin **(D)** on CES2 using CPT-11 as the probe substrate in HLMs. All data were shown as mean ± SD of triplicate assays.

**FIGURE 5:**
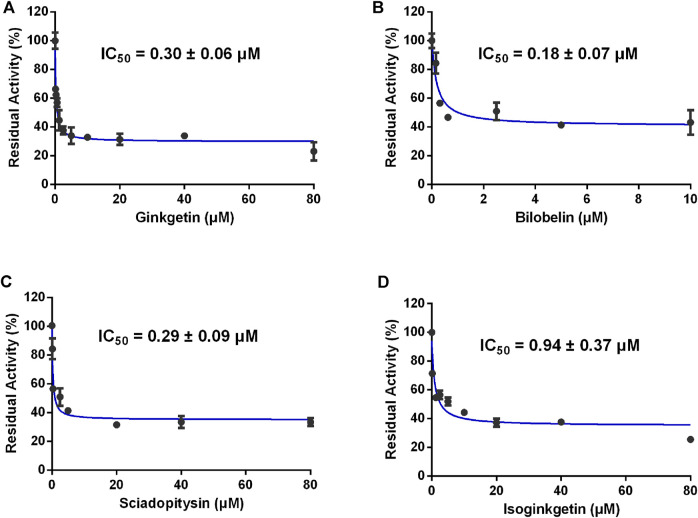
Dose-inhibition curves of ginkgetin **(A)**, bilobetin **(B)**, sciadopitysin **(C)** and isoginkgetin **(D)** on CES2 using CPT-11 as the probe substrate in HIMs. All data were shown as mean ± SD of triplicate assays.

Furthermore, the inhibitory effects of four biflavones on CES2-catalyzed irinotecan hydrolysis were also investigated in HLMs (200 μg/ml, final concentration). As listed in Table 1, four biflavones could strongly inhibit CES2-catalyzed irinotecan hydrolysis in a dose-dependent manner (Figure 4). The apparent IC_50_ values of these four biflavones against CES2-catalyzed irinotecan hydrolysis in HLMs were also determined as 1.95, 0.64, 2.86 and 0.44 μM respectively (Figure 4; Table 1), which were much lower than that inhibition of LPA on CES2-catalyzed irinotecan hydrolysis (IC_50_ = 5.35 μM, Supplementary Figure S1). Notably, owing to the extremely low hydrolytic rate of irinotecan in HLM, the high concentration of microsomal protein was used for such inhibition assay which caused larger IC_50_ values of four tested biflavones against CES2-catalyzed irinotecan hydrolysis in HLMs. The higher IC_50_ value of four biflavones on CES2-catalyzed irinotecan hydrolysis in HLMs could be partially attributed to the high binding rates of these biflavones on the microsomal protein, which led to the low free concentrations of these tested biflavones in the incubation system (Austin et al., 2002). Furthermore, the inhibitory effect of four biflavones on CES2-mediated irinotecan hydrolysis was also investigated in HIMs. As listed in Figure 5, four biflavones could strongly inhibited CES2-mediated irinotecan hydrolysis in HIMs, with the apparent IC_50_ value of 0.3, 0.18, 0.29 and 0.94 μM, respectively. These results suggest that the biflavones from *Ginkgo biloba* are potent CES2 inhibitors against CES2-catalyzed hydrolysis of both optical substrate and substrate drug (irinotecan), which encourages us to further investigate the specificity and inhibitory mechanisms of these four biflavones against CES2.

**TABLE 1:** The inhibition parameters of four biflavones from *Ginkgo biloba* against CES2-catalyzed CPT-11 hydrolysis.

Compound	Enzyme source	Microsomal protein concentration (μg/ml)	Substrate	IC_50_ (μM)	*f*_*u*_ ^a^	*f*_*u*_ × IC_50_ (μM)
Ginkgetin	HLMs	200	CPT-11	1.95 ± 0.25	0.43	0.84
Bilobetin	HLMs	200	CPT-11	0.64 ± 0.05	0.51	0.33
Sciadopitysin	HLMs	200	CPT-11	2.86 ± 0.67	0.35	1.00
Isoginkgetin	HLMs	200	CPT-11	0.44 ± 0.08	0.43	0.19

Note: Estimate the *in vitro* fraction unbound from drug’s properties. Equations published by Austin are being used for this calculation. For microsomes (Austin et al., 2002).

^a^ $f_{u}=1/\text{MicrosConc}\times 10^{0.56\times \text{logP}-1.41}+1$

logP values of ginkgetin, bilobetin, sciadopitysin and isoginkgetin were set to 3.98, 3.71, 4.24 and 3.98, respectively.

### Isoform Specificity of Four Biflavones Toward CES2

**FIGURE 6:**
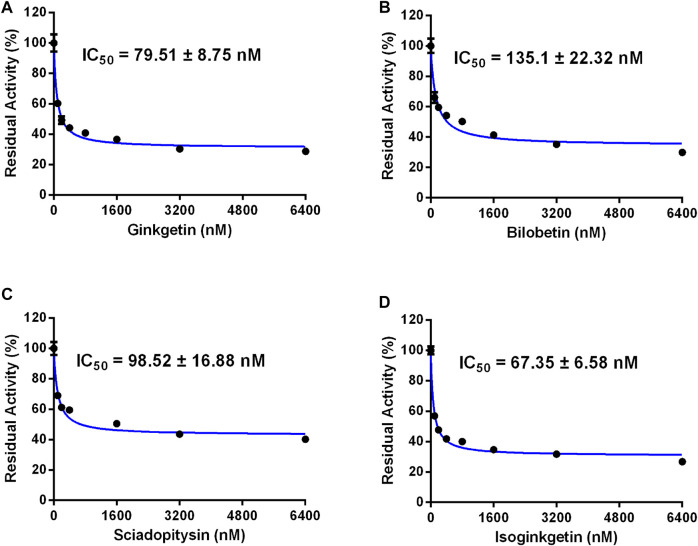
Dose-inhibition curves of ginkgetin **(A)**, bilobetin **(B)**, sciadopitysin **(C)** and isoginkgetin **(D)** on CES2 using 4-MUA as the probe substrate in recombinant CES2. All data were shown as mean ± SD of triplicate assays.

Next, the isoform specificity of these four biflavones against CES2 was assayed by using 4-MUA (a co-substrate for mammalian CES) as the substrate. As shown in Figure 6; Table 2, the inhibition of ginkgetin, bilobetin, sciadopitysin and isoginkgetin against CES2-catalyzed 4-MUA hydrolysis in recombinant CES2 were extremely potent, with the IC_50_ values as low as determined as 0.08, 0.14, 0.10 and 0.07 µM, respectively. Meanwhile, the inhibitory effects of four biflavones against CES1-catalyzed 4-MUA hydrolysis in recombinant CES1 have also been determined, and the results showed that the IC_50_ values were larger than 100 μM (Table 2). These findings clearly suggest that these four biflavones are isoform-specific inhibitors of CES2 over CES1.

**TABLE 2:** The inhibitory effects of four biflavones from *Ginkgo biloba* against CES-catalyzed 4-MUA hydrolysis.

Compound	Target enzyme	Enzyme source	Substrate	IC_50_ (μM)	Selectivity **(**CES1/CES2**)**
Ginkgetin	CES2	Recombinant CES2	4-MUA	0.08 ± 0.01	>1250
	CES1	Recombinant CES1	4-MUA	>100	
Bilobetin	CES2	Recombinant CES2	4-MUA	0.14 ± 0.02	>714
	CES1	Recombinant CES1	4-MUA	>100	
Sciadopitysin	CES2	Recombinant CES2	4-MUA	0.10 ± 0.02	>1000
	CES1	Recombinant CES1	4-MUA	>100	
Isoginkgetin	CES2	Recombinant CES2	4-MUA	0.07 ± 0.01	>1492
	CES1	Recombinant CES1	4-MUA	>100	

### Inhibitory Mechanisms of Four Biflavones Against CES2

**FIGURE 7:**
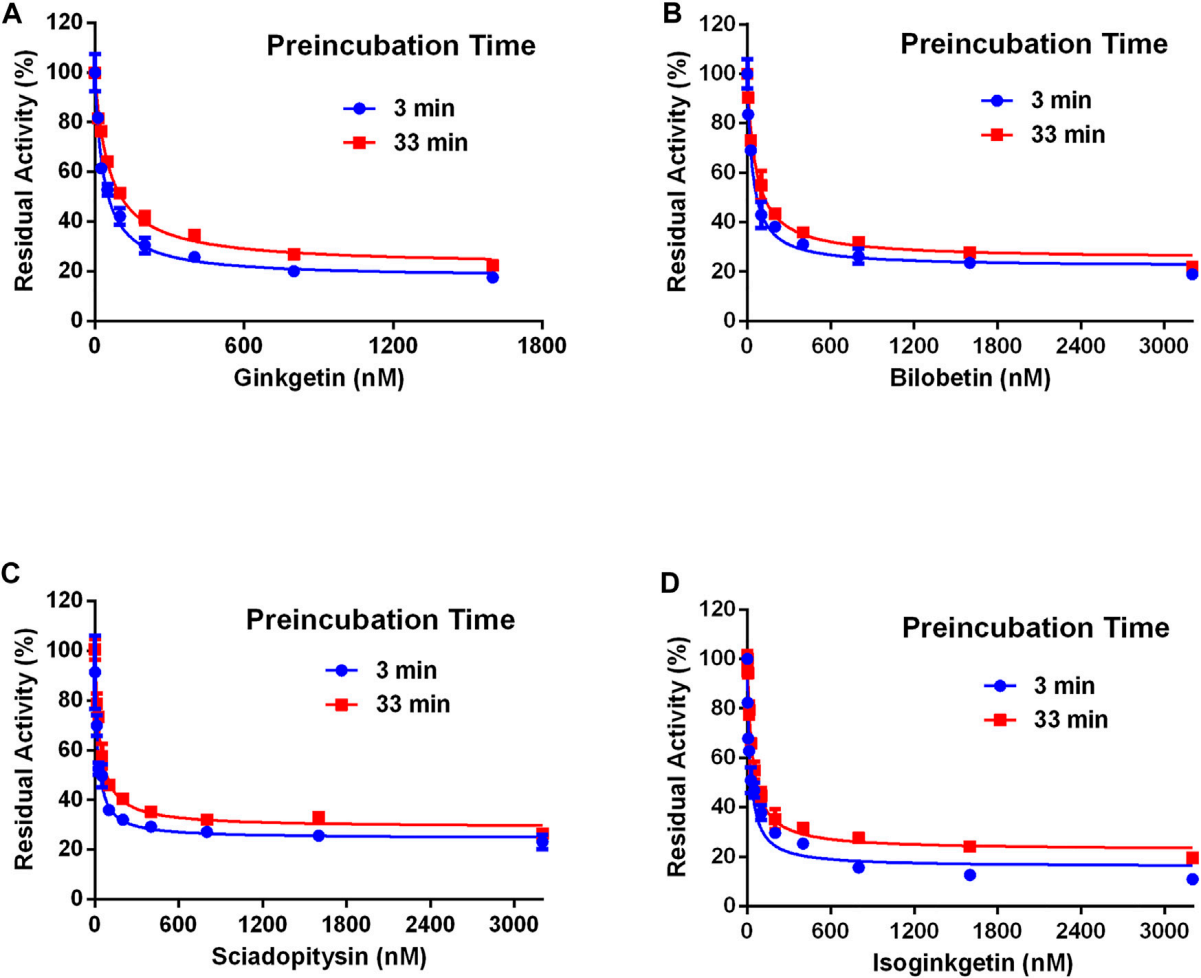
The residual activity of CES2-catalyzed hydrolysis of FD in the presence of different concentrations of biflavones from *Ginkgo biloba* at different pre-incubation times of 3 and 33 min. All data were shown as mean ± SD of triplicate assays.

**FIGURE 8:**
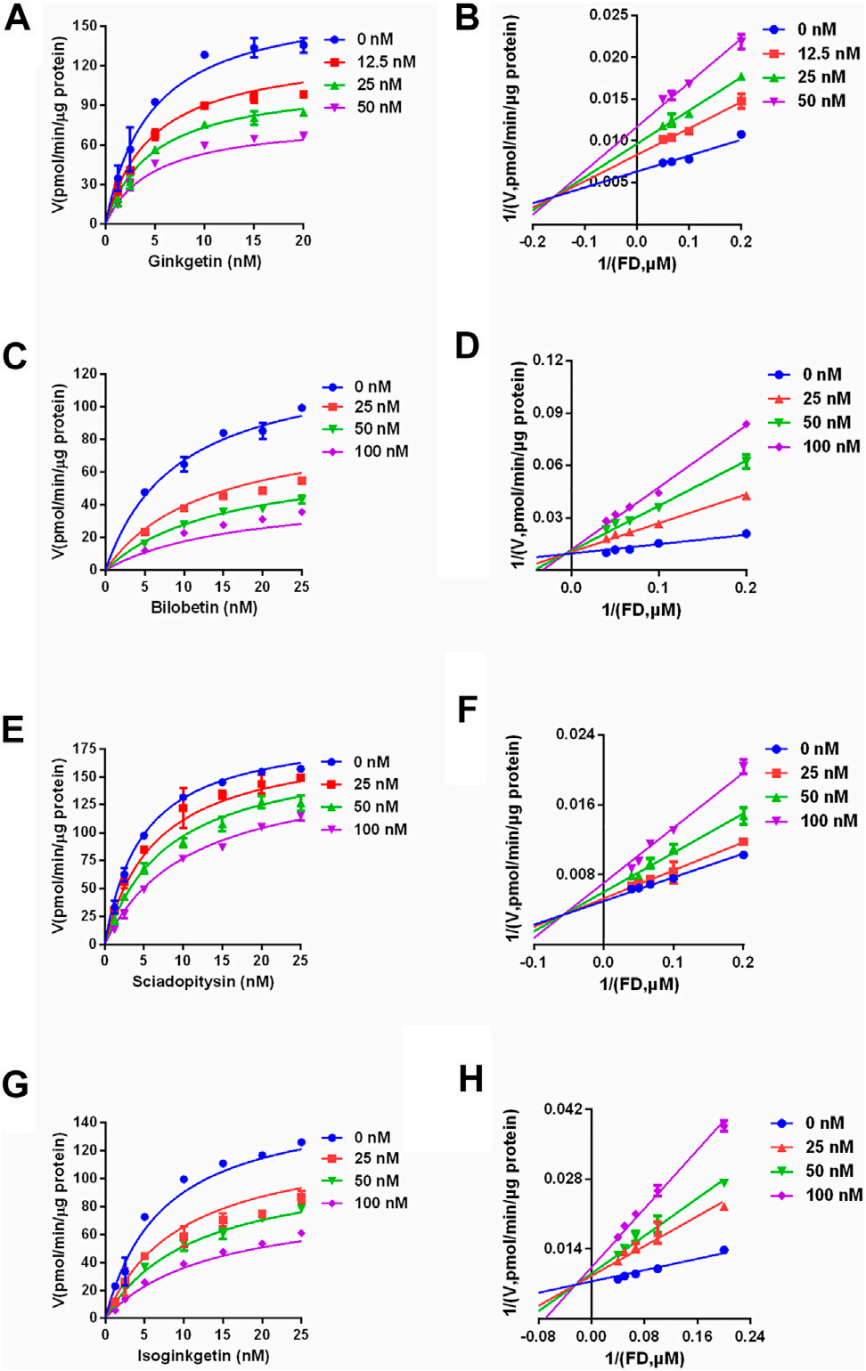
The inhibition kinetic and Lineweaver-Burk plots of ginkgetin **(A,B)**, bilobetin **(C,D)**, sciadopitysin **(E,F)** and isoginkgetin **(G,H)** against CES2-catalyzed FD hydrolysis in HLMs. All data were shown as mean ± SD of triplicate assays.

Next, the inhibitory mechanisms of four newly identified biflavone-type inhibitors were explored by performing a set of inhibition kinetic assays. Firstly, the IC_50_ shift assays were used to identify whether these biflavones were time-dependent inhibitors or reversible inhibitors against CES2 (Chen et al., 2016). As shown in Figure 7, the inhibition curves of these biflavones with different pre-incubation times (3 min or 33 min) were similar with each other, suggesting that biflavones were reversible inhibitors against CES2. Subsequently, a set of inhibition kinetic assays of these four biflavones against CES2-catalyzed FD hydrolysis were performed and the corresponding Lineweaver-Burk plots were depicted. As shown in Figure 8; Table 3, inhibition kinetic assays clearly demonstrated ginkgetin, bilobetin, sciadopitysin and isoginkgetin inhibited CES2-catalyzed FD hydrolysis in HLMs *via* a mixed inhibition manner, with *K*_*i*_ values of 32.85, 26.23, 68.77 and 32.10 nM, respectively. These findings suggest that these newly identified biflavone-type inhibitors exhibit relatively high affinity toward CES2.

**TABLE 3:** The inhibition parameters and inhibition modes on four biflavones isolated from *Ginkgo biloba* against CES2-catalyzed FD hydrolysis in HLMs.

Compound	*K*_*i*_ (nM)	Inhibition mode	Goodness of fit (*R* ^2^)
Ginkgetin	32.85	Mixed	0.97
Bilobetin	26.23	Mixed	0.96
Sciadopitysin	68.77	Mixed	0.99
Isoginkgetin	32.10	Mixed	0.98

### Molecular Mechanisms of Four Biflavones Against CES2

Comparing to the conventional docking simulations, ensemble docking approach can significantly improve the predictive performance (Amaro et al., 2018). To explore the mechanisms of the newly identified biflavones against CES2 over CES1 at the molecular level, ensemble docking simulations of bilobetin into CES2 and CES1 were performed firstly. As shown in Supplementary Figure S2; Supplementary Table S1, bilobetin could be well-docked into the catalytic cavity of CES2, with the binding energies of −9.7 kcal/mol and −10.8 kcal/mol of the top two preferred binding modes. By contrast, the lowest binding energy (−5.0 kcal/mol) of bilobetin toward CES1 was far above the CES2-bilobetin complex. These findings agreed well with the specificity of bilobetin on CES2 over CES1.

**FIGURE 9:**
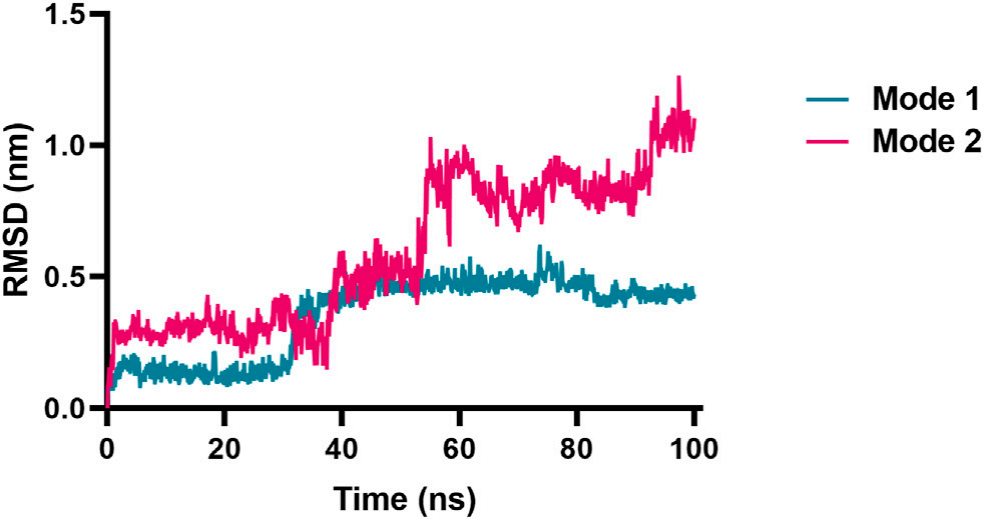
RMSD vs. time plot of bilobetin originated from the two different binding modes toward CES2 by the ensemble docking method. Mode 1 reached an equilibrium state, while mode 2 underwent large fluctuations.

**FIGURE 10:**
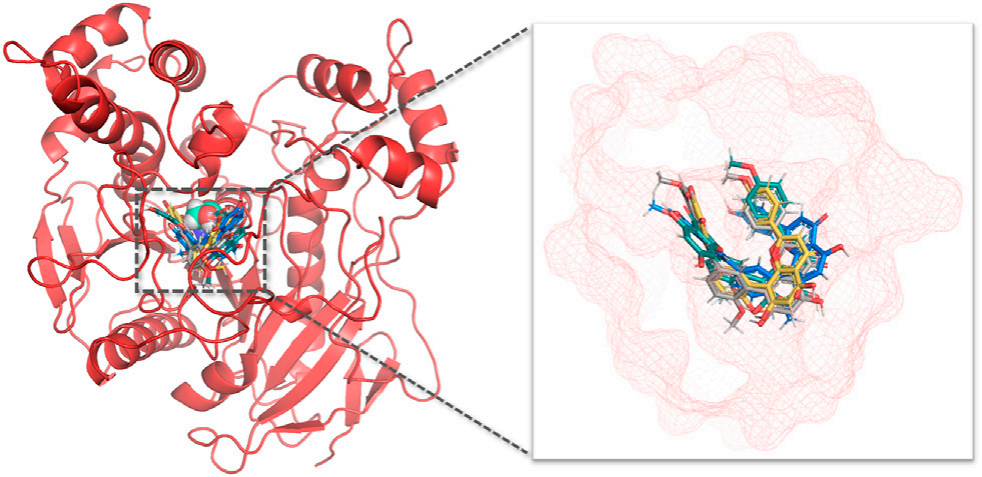
Aligned binding poses of four biflavones against CES2. The catalytic residue Ser228 was shown in cyan spheres in aerial view, ginkgetin, bilobetin, sciadopitysin, isoginkgetin were depicted with blue, yellow, gray and green, respectively. The active cavity of CES2 was displayed in red mesh.

**FIGURE 11:**
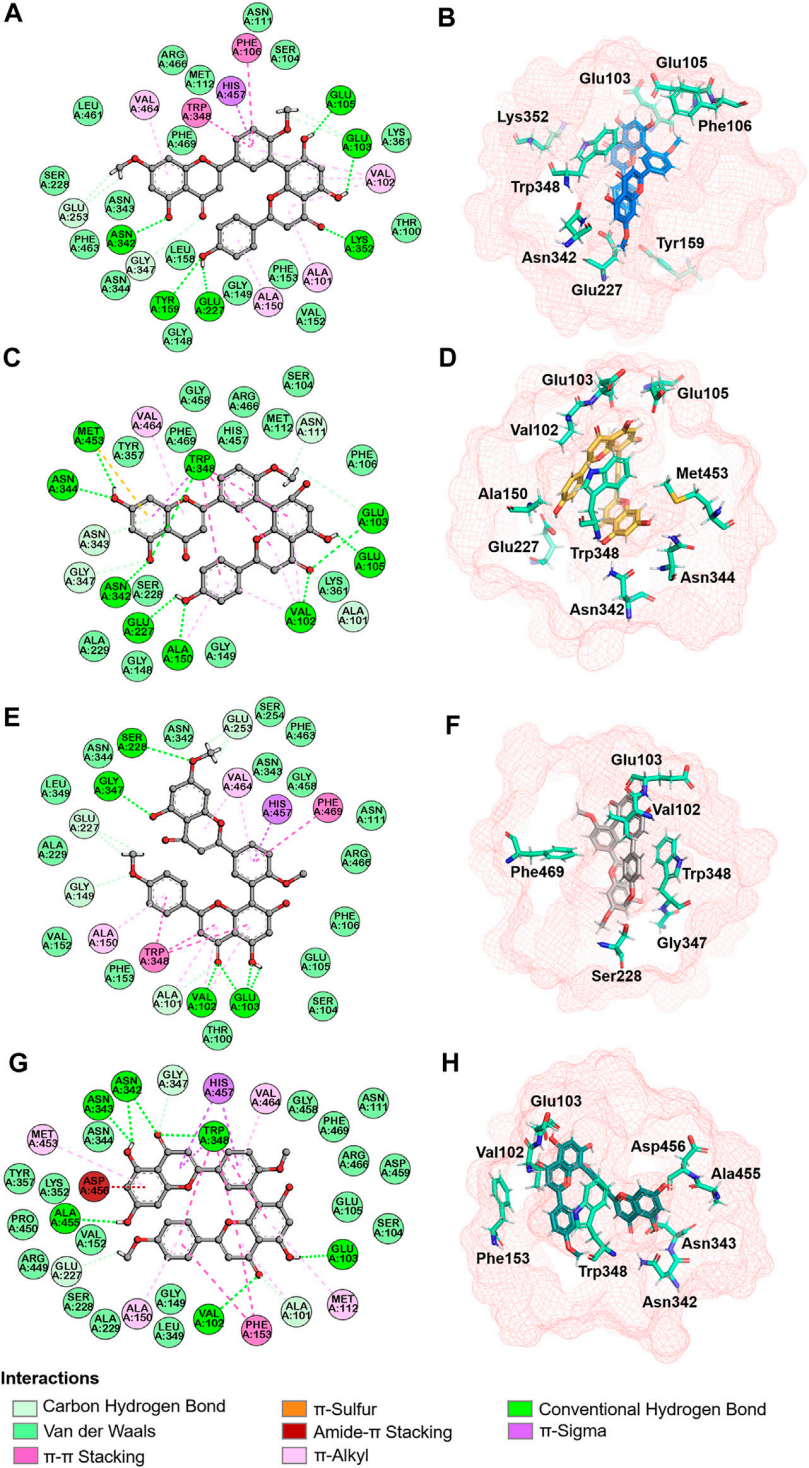
Interaction analysis and spatial arrangement with ginkgetin **(A,B)**, bilobetin **(C,D)**, sciadopitysin **(E,F)**, isoginkgetin **(G,H)** were proposed in 2D (left) and 3D (right) diagrams. Active site and pivotal residues were exhibited in red mesh and cyan sticks in stereo view.

Next, molecular dynamic simulations were used to explore the stability of the top two preferred binding modes of the CES2-bilobetin complex. As depicted in Figure 9, mode 1 could form an equilibrium state during 100 ns simulation, while mode 2 underwent large fluctuations. This finding suggested that mode 1 represented a relatively stable CES2-bilobetin complex, in which a molecule of bilobetin stably bound on the catalytic cavity of CES2. Similarly, the other three biflavones could occupy the catalytic cavity of CES2 in a similar way, which could explain why these biflavones exhibited potent CES2 inhibition activity (Figure 10). The interactions between CES2 and four biflavones were also summarized, as shown in Figure 11; Table 4. For ginkgetin, this agent created strong interactions with Glu105, Glu103, Lys352, Glu227, Tyr159 and Asn342 *via* hydrogen bonding, also interacted with Phe106 and Trp348 *via* π-π stacking. Similariy, bilobetin created strong interactions with Met453, Asn344, Trp348, Glu103, Glu105, Val102, Ala150, Glu227 and Asn342 *via* hydrogen bonding, and interacted with Trp348 and Met453 *via* π-π stacking, and π-Sulfur interaction, respectively. For sciadopitysin, four key residues (Ser228, Gly347, Val102, and Glu103) surrounding to the catalytic cavity of CES2 were involved to form hydrogen bonds with this agent, while this agent could also interact with Phe469 and Trp348 *via* π-π stacking. Isoginkgetin created strong interactions with a panel of residues (Asn343, Asn342, Trp348, Glu103, Val102, Ala455) in CES2 *via* forming hydrogen bonds, while this agent also interacted with Trp348 and Phe153 *via* π-π stacking, and with Asp456 *via* amide-π stacking. Notably, Trp348 could interact with all tested biflavones *via* π-π stacking, indicating that this residue may play a crucial role in binding on CES2. In addition, we discussed the relationship of virtual screening scores and activities against 13 representative structures obtained from clustering of molecular dynamics simulations (Supplementary Figures S3, S4). As shown in Supplementary Table S3, the overall effect was not ideal, the highest Spearman R coefficient was 0.1474. Evaluating the best binding energies and average binding energies of total structures, the former approached the optimal coefficient. In summary, the 13 receptor conformations are inadequate to capture a more diverse panel of the 120 compounds. Hence, conformations in an ensemble should endow disparate weights and more precise scoring methods are required for virtual screening of hCES2 inhibitors.

**TABLE 4:** The detailed interactions between four biflavones and CES2.

Compounds	Hydrogen bond	π-π stacking	Amide-π stacking	π-Sulfur
Ginkgetin	Glu105, Glu103, Lys352, Glu227, Tyr159, Asn342	Phe106, Trp348	\	\
Bilobetin	Met453, Asn344, Trp348, Glu103, Glu105, Val102, Ala150, Glu227, Asn342	Trp348	\	Met453
Sciadopitysin	Ser228, Gly347, Val102, Glu103	Phe469, Trp348	\	\
Isoginkgetin	Asn343, Asn342, Trp348, Glu103, Val102, Ala455	Trp348, Phe153	Asp456	\

As one of the most popular used herbal products, the extract from *Ginkgo biloba* leaves has been widely used for preparing dietary supplements and herbal medicines in both Eastern and Western countries. Over the past few decades, many studies around the world have demonstrated that *Ginkgo biloba* extract and its major constitutes may trigger clinically relevant herb-drug interactions *via* inhibition on drug-metabolizing enzymes (Wang et al., 2019). For example, *Ginkgo biloba* extract significantly inhibited a panel of human P450 enzymes, including CYP3A4 and CYP2C9. It has been reported that *Ginkgo biloba* extract may trigger clinically relevant herb-drug interactions (HDI) and bring bleeding or other side effects, when this herb is co-administrated with warfarin, an anticoagulant agent that is metabolized by CYP3A4 and CYP2C9 (Yang et al., 2006). These findings suggest that *Ginkgo biloba* extract and its major constitutes are able to modulate the pharmacokinetic properties of co-administrated drugs *via* modulating the function of drug-metabolizing enzymes. Considering that *Ginkgo biloba* extract are frequently used over the world and the recommended daily dose is very high (12 g/d), of which total biflavones were about 48.9 mg/d. The metabolic interactions of *Ginkgo biloba* ingredients with human drug-metabolizing enzymes and the potential risks should be carefully investigated (Qian and Markowitz, 2020).

Although the metabolic interactions of *Ginkgo biloba* ingredients with CYPs or UGTs have been investigated, the inhibitory effects of *Ginkgo biloba* ingredients on CES, an important class of non-CYP enzymes participating in hydrolysis of ester drugs, have not been studied yet. In this study, the inhibitory effects of abundant biflavones from *Ginkgo biloba* on CES2 were carefully investigated for the first time. Our finding clearly demonstrated that the biflavones isolated from *Ginkgo biloba* are potent and highly specific inhibitors of CES2 over CES1. Considering that CES2 plays crucial roles in hydrolytic metabolism of a wide range of therapeutic agents bearing ester or amide bond(s), much attention should be paid when *Ginkgo biloba* extract or *Ginkgo biloba*-containing dietary supplements are co-administrated with CES2 substrate-drugs. For example, CES2 can hydrolyze the antitumor drug LY2334737 (Pratt et al., 2013), the prodrug of Gemcitabine, potent inhibition of CES2 may impair the antitumor potency of LY2334737. Besides, cocaine can be hydrolyzed to its inactive metabolite by CES2 (Wu et al., 2003), strong inhibition of CES2 may slow down the metabolic clearance of these substrate-drugs *in vivo*, and then modulate its treatment outcomes.

Taking into account that intestinal CES2 is one of the key targets involving in CPT-11 induced life-threatening diarrhea, *Ginkgo biloba* leaves extract or *Ginkgo biloba*-related dietary supplements can be used for ameliorating such intestinal toxicity *via* strong inhibition on intestinal CES2. In this study, our results clearly demonstrated that the biflavones isolated from *Ginkgo biloba* displayed potent inhibitory effects against CES2, which might partially block the over-accumulation of SN-38 in the small intestine and then ameliorate CPT-11 associated life-threatening diarrhea. In view of the tissue distribution of two CES isoforms (CES2 is abundantly expressed in the small intestine but CES1 is abundantly expressed in the liver), as well as the poor oral bioavailability of the biflavones in *Ginkgo biloba*, it is easily conceivable that the local exposure of these biflavones will be much higher than that in the liver, following oral administration of *Ginkgo biloba* leaves extract. It should be noted that CES2 inhibitors may affect CPT-11 hydrolysis in the liver, which in turn, reducing the plasma exposure and the *in vivo* anti-tumor efficacy of SN38. Thus, an ideal CES2 inhibitor is expected to reduce SN-38 formation in the intestinal tract, but does not affect the hydrolysis of CPT-11 in the human liver. In this study, our findings demonstrated that these biflavones displayed very high specificity toward CES2 over CES1. In these cases, the intestinal CES2 is more likely inhibited by these biflavones but CES1 (a known serine hydrolase responsible for lipid metabolism) in the liver is hardly inhibited by these natural products.

From the perspective of drug development, biflavones are promising lead compounds for the development of novel anti-diarrhea agents for ameliorating irinotecan-induced intestinal toxicity *via* targeting CES2, owing to these compounds display potent CES2 inhibition activities, excellent isoform-specificity, good safety profiles and high local exposure in the gastrointestinal tract. Furthermore, it has been reported that the biflavones isolated from *Ginkgo biloba* leaves possess a range of bioactivities (such as anti-oxidative, anti-inflammatory), which are also beneficial for the patients with irinotecan-induced intestinal toxicity. In near future, to develop more efficacious biflavone-type CES2 inhibitors, the naturally occurring biflavones could be further modified by the medicinal chemists, to improve the CES2 inhibition potency, safety, and drug-likeness properties (as oral drugs). It is well-known that the biflavones are abundantly distributed in a range of medicinal plants (such as *Ginkgo biloba, St. John’s wort*) (van Beek and Montoro, 2009; Hou et al., 2019), while the scheme for total synthesis of biflavones has also been reported (Li et al., 1997; Ndoile and van Heerden, 2013). Thus, the structurally diverse biflavones could be easily obtained for the structure-activity relationship studies and the pharmacological assays. Structurally, the biflavones isolated from *Ginkgo biloba* leaves bear several phenolic groups, in which the C-7 phenolic group plays a crucial role CES2 inhibition (*via* forming a hydrogen bond with the residues in the catalytic cavity of CES2). Thus, the key interactions between the C-7 phenolic group in these biflavones and the catalytic cavity of CES2 should be conserved in the development of novel biflavone-type CES2 inhibitors.

## Conclusion

In summary, this study reported that four biflavones isolated from *Ginkgo biloba* displayed potent CES2 inhibition potency and high specificity over CES1 (>1000-fold). These biflavones showed potent inhibition potentials CES2-catalyzed hydrolysis of various substrates, including the CES2 substrate-drug irinotecan, while their inhibition potentials were more potent than that of loperamide (a marketed anti-diarrhea agent used for alleviating irinotecan-induced intestinal toxicity). Inhibition kinetic analyses showed that ginkgetin, bilobetin, sciadopitysin and isoginkgetin potently inhibited CES2-meidated FD hydrolysis *via* a reversible and mixed inhibition manner, with *K*_*i*_ values of less than 100 nM. Furthermore, ensemble docking and molecular dynamics give deeper insights into the molecular mechanisms of these newly identified CES2 inhibitors. All these findings clearly demonstrated that the biflavones isolated from *Ginkgo biloba* were potent and reversible CES2 inhibitors, which offered several promising lead compounds for the medicinal chemists to develop novel CES2 inhibitors for some specific purposes, such as modulating the pharmacokinetic profiles of CES2-substrate drugs and alleviating irinotecan-induced diarrhea.

## Data Availability

The raw data supporting the conclusion of this article will be made available by the authors, without undue reservation.

## Abbreviations

CES, carboxylesterases; CES2, human carboxylesterases 2; CES1, human carboxylesterases 1; FD, fluorescein diacetate; CPT-11, Irinotecan; LPA, loperamide; DMSO, dimethyl sulfoxide; HLMs, human liver microsomes; HIMs, human intestinal microsomes; IC50, Half the maximum inhibitory concentration; Ki, Inhibition constant; PBS, phosphate buffered saline; SN-38, 7-Ethyl-10-hydroxy-camptothecin; 4-MUA, 4-methylumbelliferyl acetate; EP, eppendorf tubes; RMSDs, root-mean-square deviations; MD, molecular dynamics simulation; CYPs, cytochrome P450 enzymes; UGTs, uridine diphosphate-glucuronosyltransferases.
