# Supplementary material for: Discovery and Characterization of the Biflavones From Ginkgo biloba as Highly Specific and Potent Inhibitors Against Human Carboxylesterase 2
Source: Front Pharmacol. 2021 May 18;12:655659. doi: 10.3389/fphar.2021.655659 (PMC8167799; doi:10.3389/fphar.2021.655659)
Supplement: Supplementary file 1 [file datasheet1.docx]

**Supplementary Materials**

*for*

**Discovery and Characterization of the Biflavones from *Ginkgo biloba* as Highly Specific and Potent Inhibitors against Human Carboxylesterase 2**

Yun-Qing Song^a,b,1^, Rong-Jing He^b,1^, Dan Pu^a,b^, Xiao-Qing Guan^b^, Jin-Hui Shi^b^, Yao-Guang Li^c^, Jie Hou^c^, Shou-Ning Jia^d^, Wei-Wei Qin^b*^, Sheng-Quan Fang^a*^, Guang-Bo Ge^a,b*^

^a^ Department of Gastroenterology, Yueyang Hospital of Integrated Traditional Chinese and Western Medicine, Shanghai University of Traditional Chinese Medicine, Shanghai, 200473, China.

^b^ Institute of Interdisciplinary Integrative Medicine Research, Shanghai University of Traditional Chinese Medicine, Shanghai, 201203, China.

^c^ College of Basic Medical Sciences, Dalian Medical University, Dalian, 116044, China.

^d^ Qinghai Hospital of Traditional Chinese Medicine, Xining, China.

*Corresponding author.

E-mail address: geguangbo@dicp.ac.cn (G.-B. Ge); fsq20032003@163.com (S.-Q. Fang); [wwqin@fudan.edu.cn](mailto:wwqin@fudan.edu.cn) (W.-W. Qin)

^1^ Contributed equally to this work.

**Contents**

**Fig. S1** Dose-inhibition curves of Loperamide against CES2-catalyzed CPT-11 hydrolysis.

**Fig. S2** Two different binding modes of bilobetin required from ensemble docking. Mode 1 and mode 2 were displayed in blue and gray sticks, respectively.

**Fig. S3** 13 aligned representative hCES2 structures obtained from molecular dynamics simulation.

**Fig. S4** Spearman R rank correlation plots between the binding energies and the inhibitory activities toward 13 representative structures of hCES2 (A-M). The spearman R rank correlation plot of the best binding energy (N) and average binding energy (O) against each compound and inhibitory activity. Blue dots represent four biflavone, i.e., ginkgetin, bilobetin, sciadopitysin and isoginkgetin.

**Table S1** The inhibitory effects of more than 100 kinds of natural products on CES2.

**Table S2** The lowest binding energies of bilobetin isolated from *Ginkgo biloba* towards CES1 and CES2.

**Table. S3** Spearman R rank correlation of the binding energies of each structure and total 13 structures against inhibitory activities toward hCES2.





**Fig. S1** Dose-inhibition curves of Loperamide against CES2-catalyzed CPT-11 hydrolysis. All data were shown as mean ± SD of triplicate assays.

**
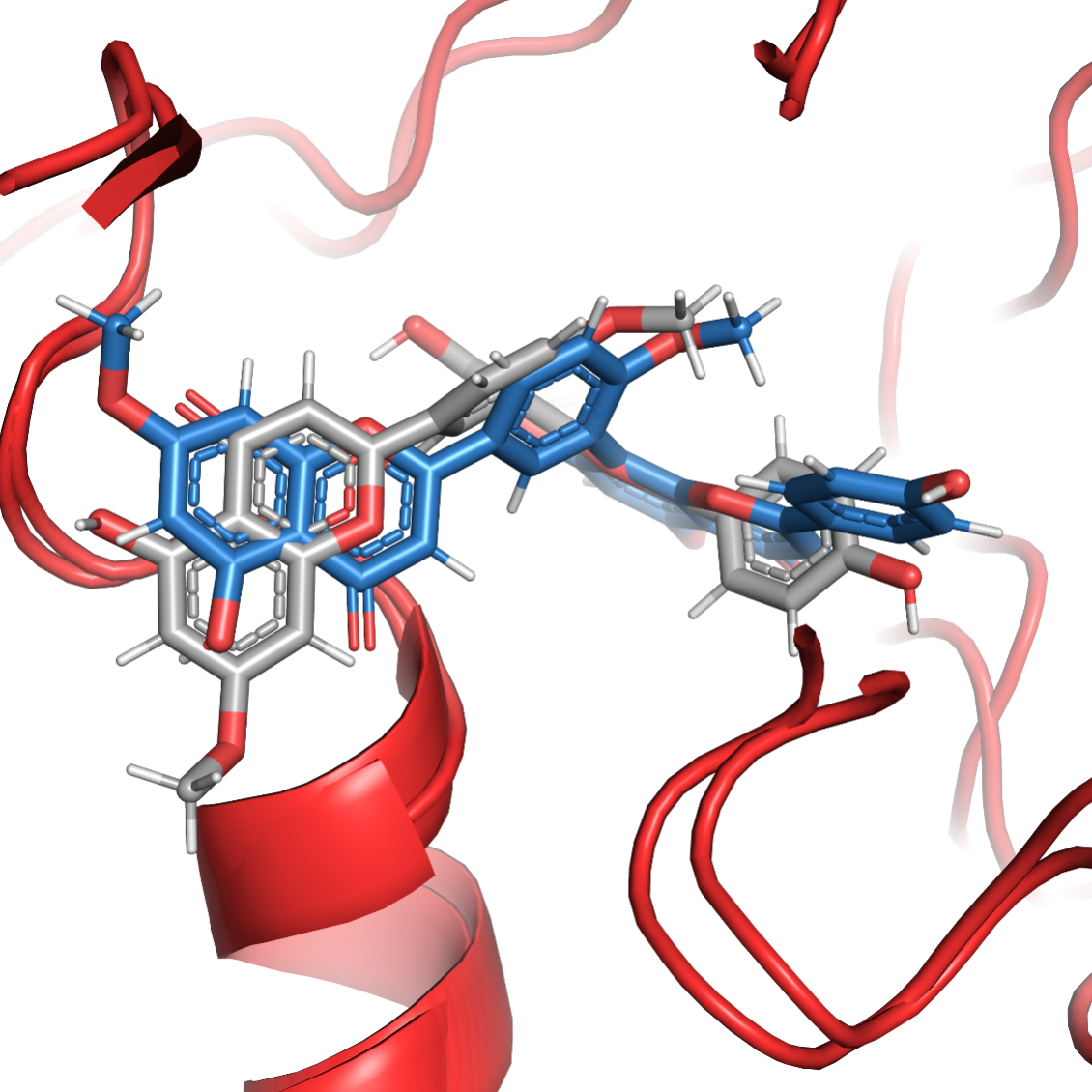
**

**Fig. S2** Two different binding modes of bilobetin required from ensemble docking. Mode **1** and mode **2** were displayed in blue and gray sticks, respectively.


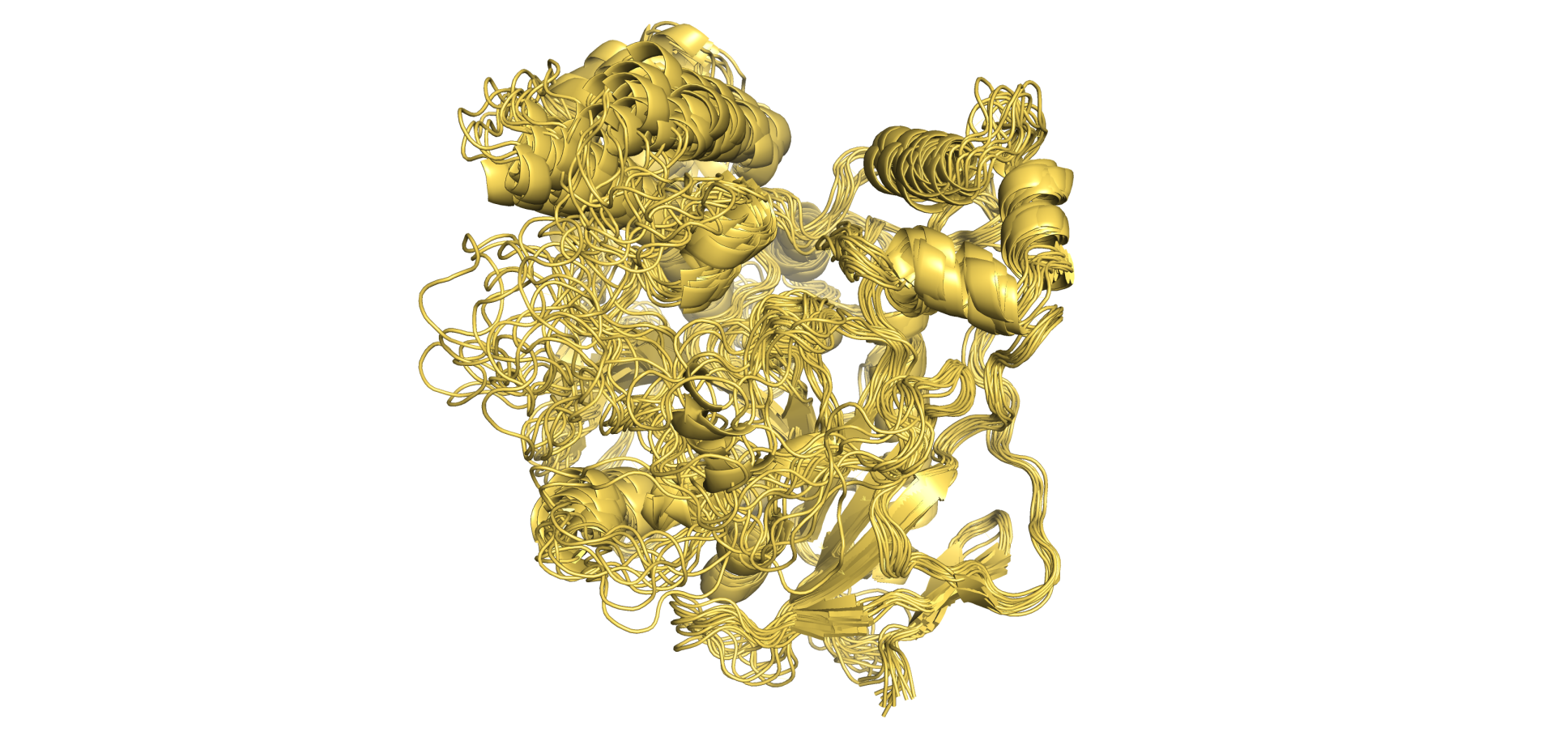


**Fig. S3** 13 aligned representative hCES2 structures obtained from molecular dynamics simulation.


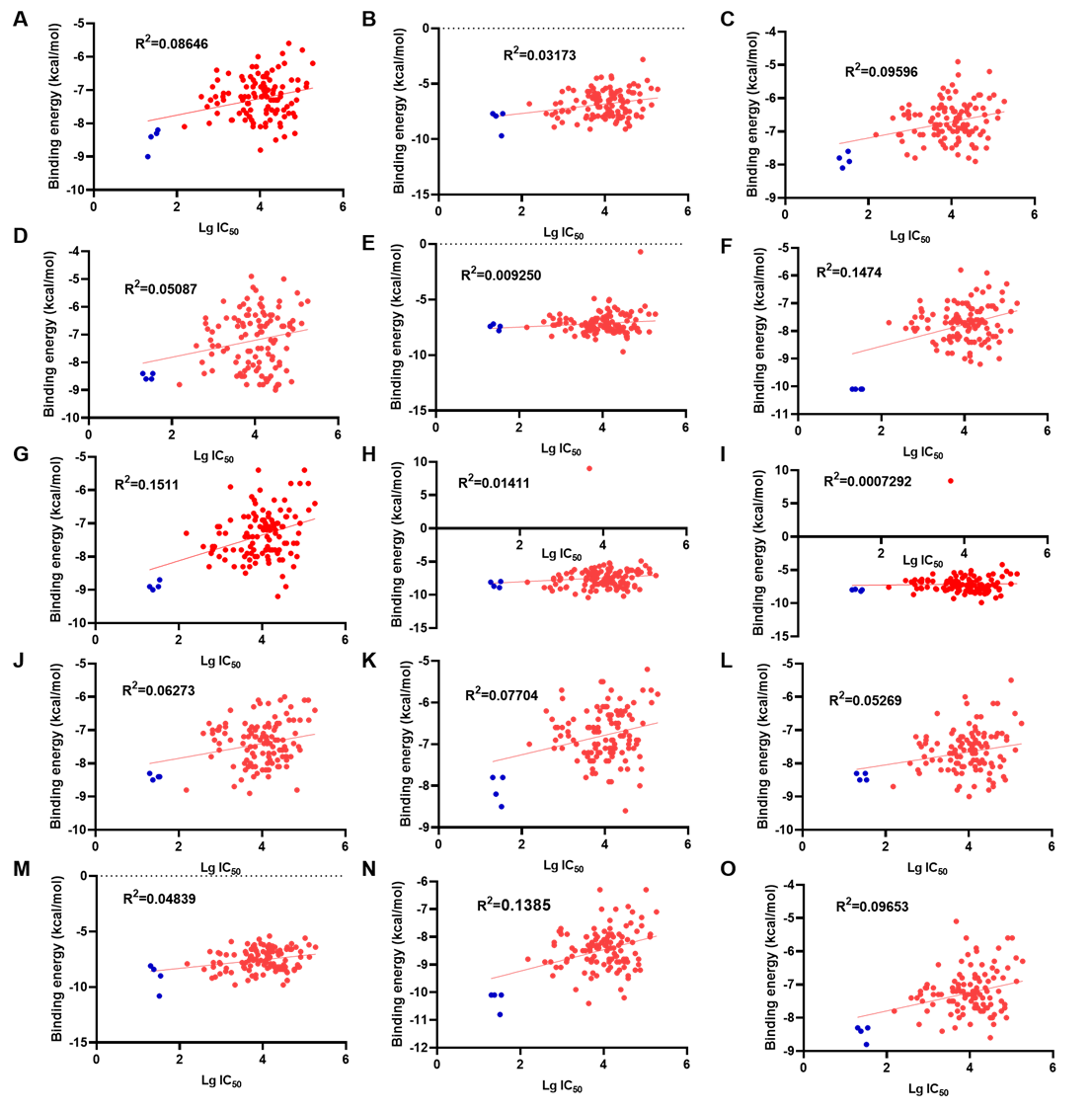


**Fig. S4** Spearman R rank correlation plots between the binding energies and the inhibitory activities toward 13 representative structures of hCES2 (A-M). The spearman R rank correlation plot of the best binding energy (N) and average binding energy (O) against each compound and inhibitory activity. Blue dots represent four biflavone, i.e., ginkgetin, bilobetin, sciadopitysin and isoginkgetin.

**Table S1**. The inhibitory effects of more than 100 kinds of natural products on CES2.

| **No.** | **Compound** | **CAS** | **CES2**  **IC_50_ (nM)** | **CES2**  **lg (IC_50_/nM)** |
| --- | --- | --- | --- | --- |
| **Biflavones** | | | | |
| 1 | Ginkgetin | 481-46-9 | 20.08 ± 1.69 | 1.30 ± 0.04 |
| 2 | Isoginkgetin | 548-19-6 | 24.20 ± 4.67 | 1.38 ± 0.08 |
| 3 | Sciadopitysin | 521-34-6 | 34.46 ± 3.15 | 1.54 ± 0.04 |
| 4 | bilobetin | 521-32-4 | 32.24 ± 3.92 | 1.51 ± 0.05 |
| **Flavonoids** | | | | |
| 5 | Glabridin | 59870-68-7 | 150 ± 20 | 2.18 ± 0.06 |
| 6 | Isorhamnetin | 480-19-3 | 3630 ± 410 | 3.56 ± 0.05 |
| 7 | Apigenin 7-glucoside | 578-74-5 | 4370 ± 1150 | 3.64 ± 0.11 |
| 8 | Genkwanin | 437-64-9 | 4730 ± 1150 | 3.67 ± 0.11 |
| 9 | 3,6-Dihydroxyflavone | 108238-41-1 | 5060 ± 830 | 3.70 ± 0.07 |
| 10 | 5,6-Dihydroxyflavone | 6665-66-3 | 3500 ± 500 | 3.54 ± 0.06 |
| 11 | Eupatilin | 22368-21-4 | 6850 ± 1140 | 3.84 ± 0.07 |
| 12 | Hispidulin | 1447-88-7 | 7640 ± 1140 | 3.88 ± 0.06 |
| 13 | Icarisid II | 113558-15-9 | 10160 ± 1440 | 4.01 ± 0.06 |
| 14 | Wogonin | 632-85-9 | 12160 ± 1540 | 4.08 ± 0.06 |
| 15 | Galangin | 548-83-4 | 12790 ± 1430 | 4.11 ± 0.05 |
| 16 | 6,7-Dihydroxyflavone | 38183-04-9 | 14570 ± 1670 | 4.16 ± 0.05 |
| 17 | Hesperetin | 520-33-2 | 18170 ± 430 | 4.26 ± 0.06 |
| 18 | Quercetin | 117-39-5 | 18850 ± 2690 | 4.28 ± 0.06 |
| 19 | Genistein | 446-72-0 | 20520 ± 2280 | 4.31 ± 0.05 |
| 20 | Oroxylin A | 480-11-5 | 20820 ± 1930 | 4.32 ± 0.04 |
| 21 | Baicalein | 491-67-8 | 21120 ± 2930 | 4.32 ± 0.06 |
| 22 | Scutellarein | 529-53-3 | 24050 ± 2990 | 4.38 ± 0.05 |
| 23 | Liquiritigenin | 578-86-9 | 31870 ± 9270 | 4.50 ± 0.13 |
| 24 | Luteolin | 491-70-3 | 34730 ± 4030 | 4.54 ± 0.05 |
| 25 | Daidzein | 486-66-8 | 57480 ± 16150 | 4.76 ± 0.12 |
| 26 | Norwogonin | 4443-09-8 | 58000 ± 5350 | 4.76 ± 0.04 |
| 27 | Chrysin | 480-40-0 | 75490 ± 6340 | 4.88 ± 0.04 |
| 28 | Nobiletin | 478-01-3 | 80490 ± 11220 | 4.91 ± 0.06 |
| 29 | Naringenin | 480-41-1 | 15480 ± 2260 | 4.19 ± 0.06 |
| 30 | 3',4'-dihydroxyflavone | 4143-64-0 | 37060 ± 7420 | 4.57 ± 0.09 |
| 31 | 1. Hydroxyflavone | 6665-83-4 | 30670 ± 3010 | 4.49 ± 0.04 |
| 32 | Nevadensin | 10176-66-6 | 132800 ± 1060 | 5.12 ± 0.01 |
| 33 | Isolicoflavonol | 94805-83-1 | 600 ± 80 | 2.78 ± 0.06 |
| 34 | Licoflavonol | 60197-60-6 | 1280 ± 90 | 3.11 ± 0.03 |
| 35 | Icaritin | 118525-40-9 | 720 ± 80 | 2.86 ± 0.05 |
| 36 | Neobavaisoflavone | 41060-15-5 | 38820 ± 8350 | 4.59 ± 0.09 |
| 37 | Bavachin | 19879-32-4 | 26490 ± 3130 | 4.42 ± 0.05 |
| 38 | Isoxanthohumol | 521-48-2 | 650 ± 70 | 2.81 ± 0.05 |
| **Chalcones** | | | | |
| 39 | Echinatin | 34221-41-5 | 3910 ± 310 | 3.59 ± 0.03 |
| 40 | Licochalcone B | 58749-23-8 | 11730 ± 1420 | 4.07 ± 0.05 |
| 41 | Isoliquiritigenin | 961-29-5 | 10720 ± 1600 | 4.03 ± 0.06 |
| 42 | Licochalcone A | 58749-22-7 | 540 ± 60 | 2.73 ± 0.05 |
| 43 | Licochalcone C | 144506-14-9 | 390 ± 40 | 2.59 ± 0.04 |
| 44 | Licochalcone D | 144506-15-0 | 940 ± 110 | 2.97 ± 0.05 |
| 45 | Isobavachalcone | 20784-50-3 | 5500 ± 410 | 3.74 ± 0.03 |
| 46 | Bavachalcone | 28448-85-3 | 6690 ± 640 | 3.83 ± 0.04 |
| 47 | Xanthohumol | 6754-58-1 | 1310 ± 140 | 3.12 ± 0.05 |
| **Alkaloids** | | | | |
| 48 | Vinblastine sulfate | 143-67-9 | 6340 ± 1120 | 3.80 ± 0.08 |
| 49 | Nonivamide | 2444-46-4 | 35050 ± 3780 | 4.54 ± 0.05 |
| 50 | Sanguinarine | 2447-54-3 | 10300 ± 1070 | 4.01 ± 0.05 |
| 51 | Piperine | 94-62-2 | 25820 ± 7130 | 4.41 ± 0.12 |
| 52 | Toddaline | 34316-15-9 | 30060 ± 11830 | 4.48 ± 0.17 |
| 53 | Coptisine chloride | 6020-18-4 | 39180 ± 14640 | 4.59 ± 0.16 |
| 54 | Rutecarpine | 84-26-4 | 30970 ± 7780 | 4.49 ± 0.11 |
| 55 | Isorhynchophylline | 6859-01-4 | 16900 ± 3020 | 4.23 ± 0.08 |
| 56 | Boldine | 476-70-0 | 6530 ± 1040 | 3.81 ± 0.07 |
| 57 | Capsaicin | 404-86-4 | 27830 ± 4910 | 4.44 ± 0.08 |
| 58 | Chelidonine | 476-32-4 | 13400 ± 5400 | 4.13 ± 0.18 |
| 59 | Coptisine | 3486-66-6 | 39180 ± 14640 | 4.59 ± 0.16 |
| 60 | Reserpine | 50-55-5 | 940 ± 120 | 2.97 ± 0.06 |
| 61 | Catharanthine | 2468-21-5 | 12850 ± 900 | 4.11 ± 0.03 |
| 62 | Uncarine E | 5171-37-9 | 5940 ± 890 | 3.77 ± 0.07 |
| 63 | Vinpocetine | 42971-09-5 | 7030 ± 750 | 3.85 ± 0.05 |
| 64 | Cyclovirobuxine D | 860-79-7 | 13330 ± 2440 | 4.12 ± 0.08 |
| 65 | Solasodine | 126-17-0 | 24200 ± 4410 | 4.38 ± 0.08 |
| **Terpenoids** | | | | |
| 66 | Euphorbia factor L3 | 218916-52-0 | 14660 ± 2900 | 4.17 ± 0.09 |
| 67 | Ginsenoside K | 39262-14-1 | 15460 ± 1560 | 4.19 ± 0.04 |
| 68 | Euphorbia Factor L1 | 76376-43-7 | 22960 ± 3090 | 4.36 ± 0.06 |
| 69 | Glycyrrhetic Acid | 471-53-4 | 69260 ± 8920 | 4.84 ± 0.06 |
| 70 | Ginsenoside Rc | 11021-14-0 | 81660 ± 18560 | 4.91 ± 0.10 |
| 71 | Ginsenoside F1 | 53963-43-2 | 37080 ± 5090 | 4.57 ± 0.06 |
| 72 | Ginsenoside Rd | 52705-93-8 | 14050 ± 1780 | 4.15 ± 0.06 |
| 73 | 1. Deoxoglycyrrhetinic acid | 564-16-9 | 6950 ± 690 | 3.84 ± 0.04 |
| 74 | Ginsenoside F2 | 62025-49-4 | 14370 ± 1590 | 4.16 ± 0.05 |
| 75 | Panaxatriol | 32791-84-7 | 14590 ± 1090 | 4.16 ± 0.03 |
| 76 | Cyclovirobuxine D | 860-79-7 | 13330 ± 2440 | 4.12 ± 0.08 |
| 77 | Ginsenoside F4 | 181225-33-2 | 45020 ± 6480 | 4.65 ± 0.06 |
| 78 | Ginsenoside Rh3 | 105558-26-7 | 5850 ± 1170 | 3.77 ± 0.09 |
| 79 | Ginsenoside Rh4 | 174721-08-5 | 62850 ± 6720 | 4.80 ± 0.05 |
| 80 | α-Boswellic acid | 471-66-9 | 2030 ± 290 | 3.31 ± 0.06 |
| 81 | β-Boswellic acid | 631-69-6 | 3140 ± 400 | 3.50 ± 0.06 |
| 82 | Ginsenoside Rh2 | 78214-33-2 | 5300 ± 530 | 3.72 ± 0.04 |
| 83 | Panaxadiol | 19666-76-3 | 3780 ± 390 | 3.58 ± 0.04 |
| 84 | Protopanaxadiol | 30636-90-9 | 1330 ± 100 | 3.12 ± 0.03 |
| 85 | Ginsenoside Rg3 | 14197-60-5 | 10370 ± 1170 | 4.02 ± 0.05 |
| 86 | Betulin | 473-98-3 | 4300 ± 480 | 3.63 ± 0.05 |
| 87 | 11-Keto-β-boswellic acid | 17019-92-0 | 68870 ± 3380 | 4.84 ± 0.02 |
| 88 | Celastrol | 34157-83-0 | 14110 ± 2560 | 4.15 ± 0.08 |
| 89 | Pachymic acid | 29070-92-6 | 14120 ± 1100 | 4.15 ± 0.03 |
| 90 | Protopanaxatriol | 34080-08-5 | 860 ± 90 | 2.93 ± 0.05 |
| 91 | Dammarenediol II | 14351-29-2 | 650 ± 100 | 2.81 ± 0.07 |
| 92 | Epibetulinic acid | 38736-77-5 | 48540 ± 8800 | 4.69 ± 0.08 |
| 93 | Betulinic acid | 472-15-1 | 23610 ± 2650 | 4.37 ± 0.05 |
| 94 | Oleanolic acid | 508-02-1 | 13500 ± 1370 | 4.13 ± 0.04 |
| 95 | Betulonic acid | 4481-62-3 | 17230 ± 1260 | 4.24 ± 0.03 |
| 96 | Corosolic acid | 4547-24-4 | 12360 ± 1170 | 4.09 ± 0.04 |
| 97 | Maslinic acid | 4373-41-5 | 25490 ± 8710 | 4.41 ± 0.15 |
| 98 | Quinatic acid | 119863-89-7 | 5300 ± 1200 | 3.72 ± 0.10 |
| 99 | β-amyrin | 559-70-6 | 4010 ± 500 | 3.60 ± 0.05 |
| 100 | Ursolic acid | 77-52-1 | 7540 ± 1450 | 3.88 ± 0.08 |
| 101 | 11-Deoxyalisol B | 155073-73-7 | 4630 ± 1300 | 3.67 ± 0.12 |
| 102 | β-amyrenone | 638-97-1 | 5000 ± 900 | 3.70 ± 0.08 |
| 103 | Oleanderolide | 32619-42-4 | 5590 ± 1530 | 3.75 ± 0.12 |
| **Lignans** | | | | |
| 104 | Schisandrin B | 61281-37-6 | 1750 ± 220 | 3.24 ± 0.05 |
| 105 | Magnolol | 528-43-8 | 910 ± 20 | 2.96 ± 0.01 |
| 106 | Honokiol | 35354-74-6 | 960 ± 100 | 2.98 ± 0.05 |
| 107 | Deoxyschizandrin | 61281-38-7 | 8060 ± 1060 | 3.91 ± 0.06 |
| 108 | Schisanhenol | 69363-14-0 | 8910 ± 1320 | 3.95 ± 0.06 |
| 109 | Schisantherin E | 64917-83-5 | 38600 ± 2500 | 4.59 ± 0.03 |
| 110 | Schisandrol A | 7432-28-2 | 105000 ± 4070 | 5.02 ± 0.02 |
| 111 | Schisandrol B | 58546-54-6 | 129000 ± 7500 | 5.11 ± 0.03 |
| 112 | Gomisin J | 66280-25-9 | 48700 ± 3300 | 4.69 ± 0.03 |
| 113 | Gomisin G | 62956-48-3 | 19500 ± 2000 | 4.29 ± 0.04 |
| 114 | Gomisin O | 72960-22-6 | 188000 ± 6800 | 5.27 ± 0.02 |
| **Others** | | | | |
| 115 | Estriol | 50-27-1 | 11270 ± 1930 | 4.05 ± 0.07 |
| 116 | Resveratrol | 501-36-0 | 12510 ± 1880 | 4.10 ± 0.07 |
| 117 | Gancaonin I | 126716-36-7 | 1720 ± 240 | 3.24 ± 0.06 |
| 118 | Psoralidin | 18642-23-4 | 2140 ± 320 | 3.33 ± 0.06 |
| 119 | Glycycoumarin | 94805-82-0 | 6750 ± 880 | 3.83 ± 0.06 |
| 120 | Shikonin | 517-89-5 | 8980 ± 520 | 3.95 ± 0.03 |
| -- | LPA (Positive inhibitor) | 34552-83-5 | 6240 ± 930 | 3.80 ± 0.06 |
| -- | BNPP (Positive inhibitor) | 645-15-8 | 490 ± 70 |  |

**Table S2**. The lowest binding energies of bilobetin isolated from *Ginkgo biloba* towards CES1 and CES2.

| **Target enzyme** | **Binding energy (kcal/mol)** |
| --- | --- |
| CES1 | -5.0 |
| CES2 | -9.7 (Mode 1) |
|  | -10.8 (Mode 2) |

**Table. S3** Spearman R rank correlation of the binding energies of each structure and total 13 structures against inhibitory activities toward hCES2.

| **Structure** | **Spearman R rank correlation** |
| --- | --- |
| S1 | 0.03173 |
| S2 | 0.09596 |
| S3 | 0.05087 |
| S4 | 0.009250 |
| S5 | 0.1474 |
| S6 | 0.1511 |
| S7 | 0.01411 |
| S8 | 0.0007292 |
| S9 | 0.06273 |
| S10 | 0.07704 |
| S11 | 0.05269 |
| S12 | 0.04839 |
| S13 | 0.08646 |
| Total structures  (best energy) | 0.1385 |
| Total structures (average energy) | 0.09653 |
